# Determinants of Childhood Anemia in India

**DOI:** 10.1038/s41598-019-52793-3

**Published:** 2019-11-12

**Authors:** Nkechi G. Onyeneho, Benjamin C. Ozumba, S. V. Subramanian

**Affiliations:** 1 Takemi Program in International Health, Department of Global Health and Population, Harvard TH Chan School of Public Health, 677 Huntington Avenue, Boston, Massachusetts USA; 2 Department of Sociology/Anthropology, University of Nigeria, Nsukka, Enugu State Nigeria; 3 Department of Obstetrics and Gynaecology, University of Nigeria, Nsukka, Enugu State Nigeria; 4 Department of Social and Behavioral Sciences, Harvard TH Chan School of Public Health, 677 Huntington Avenue, Boston, Massachusetts USA

**Keywords:** Risk factors, Ecology

## Abstract

We analyzed a sample of 112714 children from the 2015–2016 Indian National Fertility and Health Survey with available data on hemoglobin. Multinomial logistic regression models were used to establish associations between parent anemia, household characteristics and nutritional intake of children. Linear regression analysis was also conducted to see the link between the household characteristic and childhood nutritional intake on one hand and hemoglobin levels on the other hand. A number of socio-demographic factors, namely maternal age, type of residence and maternal education, as well as wealth index, among others correlate with incidence of childhood anemia. For instance, whereas 52.9% of children in the richest households were anemic, 63.2% of children in the poorest household were anemic (p < 0.001). Mean Vitamin A intake in the last six months was 0.63 (0.626–0.634) which was 0.18% of the recommended intake. Mean iron intake, from sources other than breast milk, in the last 24 hours was 0.29 (0.286–0.294) and 2.42% of the recommended daily intake. Fifty-nine percent (58.5%) of the children surveyed were anemic (Hb level: 9.75 g/dL [9.59–9.91]). Children with anemia were more prone to being iron deficient (odds ratio [OR]: 0.981 (0.961–1.001), Vitamin A deficient (OR: 0.813 (0.794–0.833)), and have lower maternal hemoglobin level (OR: 1.992 (1.957–2.027)). Combining nutritional supplementation and food-fortification programmes with reduction in maternal anemia and family poverty may yield optimal improvement of childhood anemia in India.

## Introduction

Childhood anemia remains one of the most salient nutritional disorder facing mothers and children in India despite a massive increase in private and public humanitarian efforts to promote child health in response to anemia rates reported in earlier demographic and health surveys^1–3^. The 2005/06 National Family Health Survey (NFHS) in India revealed that at least 80% of children between 12 and 23 months were anemic in India. And in children aged <5 years, 69.5% were anemic^4^. Unfortunately, recent economic development and the national anemia-control programme have not translated to major reduction in the occurrence of anemia in India as shown in the 2015 figures^5^. The 2015 demographic health survey show only 11 percentage point decrease from 69.5% to 58.5% childhood anemia in India, still making it endemic. The elimination of iron deficiency anemia in children is a public-health priority, given the association of anemia with impaired cognitive and psychomotor development^6,7^.

Iron deficiency, linked to low nutritional iron consumption is one of the critical causes of childhood anemia in India^8^. Other critical factors, equally associated with childhood anemia here, include vitamin deficiencies, especially folate, vitamin B12 and A, infections with malaria parasite, hookworm, and hemoglobinopathies^8–10^. In a study of childhood anemia in rural India, Pasricha *et al*. suggested that the level of Hemoglobin was principally linked with the status of iron in children. it also revealed that maternal hemoglobin level, family wealth, and food insecurity were equally critical^11^.

It is thus surmised that childhood anemia in India result could be explained by the low micronutrient (especially iron) levels, compounded some socioeconomic conditions. To effectively address situation, health providers would require a comprehensive understanding of these root causes of anemia. There is currently a dearth of published literature describing the drivers of childhood anemia in children less than 5 years in India. Pasricha *et al*. documented the determinants of childhood anemia in two rural districts of Karnataka India, but this was to the exclusion of the urban and urban slums in India^11^. Using a nationally representative data, with comprehensive and objective measures of anemia as well as a range of socioeconomic factors, we assessed the relative and joint contribution of these factors in predicting childhood anemia in India.

## Methods

### Data

The Indian Demographic and Health Survey (DHS) 2015–2016 provided data for this analysis. The DHS Program which operates in over 80 low/middle income countries^12^. The DHS used standardized questionnaires to collect information on child health, socioeconomic conditions, household characteristics and other indicators from nationally representative samples of children less than 5 years. The 2015–2016 NFHS used a multistate, stratified cluster sampling procedure which has been described elsewhere^13^. There were a total of 217324 children aged <5 years in the DHS.

In this survey, anemia testing was performed by measuring hemoglobin (Hb) concentration from a drop of blood obtained from by finger prick. Children with severe anemia refers to the proportion of children under age 5 classified as having severe (below 7.0 g/dl) anemia. Children with moderate anemia refer to proportion of children under age 5 classified as having moderate (7.0–9.9 g/dl) anemia. Children with mild anemia refer to proportion of children under age 5 classified as having mild (10.0–10.9 g/dl) anemia. Children with any anemia referred to proportion of children under age 5 classified as having any anemia. For this analysis, we selected a sample of 112714 children from the NFHS with available data on hemoglobin and anemia.

### Study population and sample size

The study population constitutes a nationally representative cross-sectional sample of singleton children aged 0–59 months alive at the time of survey and born after January 2010/2011.

### Outcome

The primary outcome was child’s Hb level (in g/dl) and grades of anemia defined as mild (10–10.9 g/dl), moderate (7–9.9 g/dl), and severe (<7 g/dl). For analyses purposes, the moderate and severe categories were combined. Blood testing was conducted by trained personnel, who were part of the survey team. The finger prick tests were carried out in the homes of the children and their mothers, and blood samples were tested immediately using a portable HemoCue analyzer (201+).

### Exposures

Maternal Hb was measured as in children using a finger prick blood sample. Grades of anemia were defined as mild (10–11.9 g/dl in women and 10–10.9 in pregnant women), moderate (7–9.9 g/dl in women), and severe (<7.0 g/dl in women). Similar to children, the analysis of maternal anemia combined all types of anemia.

### Covariates

We included a number of household level socioeconomic covariates as well as maternal characteristics which may be associated with child Hb levels. The sample frequency distribution, and proportion of anemic children across the characteristics are given in Table 1.

**Table 1:** Proportion of children with anemia (Hemoglobin <11 g/dl) and socio-demographic factors.

Characteristics	Categories	Number	% Anemic (95% CI)	χ^2^	P value
Maternal age	15–19	3988	64.8 (0.633–0.662)	847.46	<0.001
	20–24	60175	61.6 (0.612–0.620)		
	25–29	82188	56.9 (0.566–0.573)		
	30–34	41184	54.7 (0.542–0.552)		
	35–39	16035	52.6 (0.519–0.534)		
	40–44	4608	52.9 (0.514–0.543)		
	45–49	1317	53.1 (0.504–0.558)		
Residence	Urban	49826	55.2 (0.547–0.556)	148.34	<0.001
	Rural	159669	58.3 (0.580–0.585)		
Maternal education	No education	65916	64.3 (0.639–0.646)		
	Primary	30664	58.3 (0.578–0.589)		
	Secondary	93929	54.0 (0.537–0.543	2138.287	<0.001
	Higher	18986	50.1 (0.494–0.508		
Maternal Literacy	Cannot read	74325	63.8 (0.634–0.641)		
	Can read only part	13914	57.4 (0.565–0.582)		
	Able to read	119748	53.6 (0.533–0.539)	1953.41	<0.001
Wealth index	Poorest	55133	63.2 (0.628–0.636)		
	Poorer	49361	57.6 (0.572–0.581)	1196.866	<0.001
	Middle	41853	56.2 (0.557–0.567)		
	Richer	35001	53.6 (0.533–0.544)		
	Richest	14880	52.9 (0.523–0.535		
Sex of child	Male	109087	57.5 (0.572–0.578)	0.156	0.348
	Female	100408	57.6 (0.573–0.579)		

### Analysis

Linear regression analysis based on continuous variables retained maximum information^11,14^. For this reason, hemoglobin level, rather than anemia was analyzed in this study as the outcome variable for the linear regression model, as the threshold for anemia has age and ethnic ambiguities^15^. Nutritional intake was viewed as categorical variable. The survey collected information on daily intake using the 24 hours maternal recall of dietary intake measurement techniques recommended by the WHO to assess child dietary intake. This relied on self-report

Risk factors and outcomes links were first assessed by using univariate correlation. Multiple-regression models were then iteratively developed. Variables were retained where the P value for their coefficient remained ≤0.05.

### Ethical consideration

Ethical approval for the National Family Health Survey (NFHS-4), 2015–16, was obtained from the Indian ethics review board and the Ministry of Health and Family Welfare, India.

## Results

### Socio-demographic characteristics and childhood anemia

The India demographic and health survey (DHS) results for 2015/16 show an eleven percentage point difference in childhood anemia from the situation 10 years earlier (2005/2006). The proportion of children with any anemia now stands at 58.5% compared to 69.5% in the 2005/2006 DHS. The proportions with severe and moderate anemia dropped to 1.6% and 29.25 respectively. However, the proportion with mild anemia rose to 27.3% from 26.3% in 2005/06.

A number of socio-demographic factors, namely maternal age, type of residence and maternal education correlate with incidence of anemia in the children (Table 1). Other factors include maternal ability to read, household wealth index. Children born to younger mothers aged 15 to 29 years tended to be more anemia (p < 0.001). Children in rural communities tended to be more anemic than their counterparts in the urban communities (p < 0.001) and children of less educated mothers were more anemic (p < 0.001). Mothers with reading abilities had fewer anemic children compared to their counterparts who could not read (p < 0.001). There were fewer cases of childhood anemia in the wealthier households. For instance, whereas 52.9% of children in the richest households were anemic, 63.2% of children in the poorest household were anemic (p < 0.001).

### Nutritional intake and childhood anemia

The mean nutritional intake for the children in the survey is shown in Table 2. Mean Vitamin A intake in the last six months was 0.63 (0.626–0.634) which was 0.18% of the recommended intake. On the other hand, the intake of Vitamin A in the last 24 was 1.31 (1.306–1.313) and 0.37% of the recommended daily intake. The mean iron intake from the consumption of other than breast milk in the last 24 hours was 0.29 (0.286–0.294) and 2.42% of the recommended daily intake.

**Table 2:** Mean Nutrient intake from non-breast milk sources.

Nutrients	Mean (95% CI)	Required Daily Intake (RDI)	Mean intake % of RDI (95% CI)
Vitamin A (at 24 months)	1.31 (1.306–1.313)	350	0.374286
Iron	0.29 (0.286–0.294)	12	2.416667
Vitamin A (at 12 months)	0.63 (0.626–0.634)	350	0.18

The hemoglobin levels and organic factors linked to hemoglobin levels are shown in Table 3. Slightly more than Fifty-eight percent (58.5%) of the children surveyed were anemic (Hb level: 9.75 g/dL [9.59–9.91]). Childhood iron deficiency was seen in 76.3% (66.5–76.1%) Anemic children were more prone to iron deficiency (odds ratio [OR]: 0.981 (0.961–1.001), Vitamin A deficiency (OR: 0.813 (0.794–0.833)), maternal hemoglobin level (OR: 1.992 (1.957–2.027)).

**Table 3:** Proportion of children with anemia (Hemoglobin <11 g/dl) and associated conditions.

Associated Factors	% (95 CI)	Anemic	
		% (95% CI yes)	Odds Ratio no*
Iron deficiency	76.3 (0.761–0.765)	57.6 (0.574–0.579)	0.981 (0.961–1.001)
Vitamin A deficiency	80.0 (0.798–0.801)	58.3 (0.581–0.586)	0.813 (0.794–0.833)
Worm infestation	27.3 (0.271–0.275)	58.7 (0.584–0.589)	0.855 (0.839–0.872)
Inflammatory disease	9.1 (0.080–0.092)	64.2 (0.635–0.649)	1.362 (1.321–1.406
Common Infections	17.2 (0.171–0.174	58.9 (0.058–0.060)	1.073 (1.049–1.098)
Maternal hemoglobin level	56.1 (0.559–0.562)	64.9 (0.647–0.652)	1.992 (1.957–2.027)

*Odds Ratio means this is the outcome per unit increase in the value of exposure. In other words, the exponential function of the logistic regression coefficient is the odds ratio associated with a unit increase in the exposure.

### Association with childhood anemia

Anemia was positively associated with vitamin intake in children (coefficient: 0.135 [0.849–0.899]; *P* < 0.001); worm infestation (coefficient: 0.137 [0.848–0.896]; p < 0.001) and iron intake (coefficient 0.110 [1.084–1.149]; p < 0.001). It was higher in boys (*t* = 0.027 (1.004–1.052) (p = 0.019). The univariate logistic regression analysis further revealed that levels of childhood anemia were negatively associated with the age and educational attainment of mothers (Table 4). Results of the multiple regression analysis revealed that childhood hemoglobin levels were primarily linked to their level of nutritional consumption, maternal hemoglobin levels and household wealth. Age and inflammatory diseases in children were inversely associated hemoglobin in the children (Table 5). Comparing some key indices of hemoglobin revealed that height and weight impact more on hemoglobin level than nutritional status (Table 6). On the other hand, level of vitamin A intake affected level of hemoglobin.

**Table 4:** Logistic regression coeficient between childhood anemia and other conditions.

Childhood Conditions	Logistic Regression coefficient (95% CI)	P value
Vitamin Deficiency	0.135 (0.849–0.899)	0.000
Iron Deficiency	0.110 (1.084–1.149)	0.000
Worm Infestation	0.137 (0.848–0.896)	0.000
Had Fever	0.188 (1.129–1.291)	0.000
Had Cough	0.164 (0.796–0.904)	0.000
Inflammatory Infection	0.285 (1.275–1.388)	0.000
Common Infections	0.006 (0.926–1.094)	0.882
Low Maternal Hemoglobin	0.657 (1.886–1.975)	0.000
Male Child	0.027 (1.004–1.052)	0.019
Wealthy Household	0.011 (0.963–1.015)	0.402
Able To Read	0.011(0.769–1.328)	0.939
Maternal Education (Above primary)	−0.092 (0.881–0.944)	0.000
Living In Urban Community	0.010 (0.983–1.038)	0.477
Younger Mothers (15–29 Years)	−0.317 (1.337–1.411)	0.000

**Table 5:** Multiple Regression Model of Associates with Childhood Hemoglobin.

Associate Factors	Coefficient B	95.0% Confidence Interval for B		Standardized Coefficients	Sig.
		Lower Bound	Upper Bound		
(Constant)	21.958	18.001	25.915		0.000
Vitamin A Deficiency	−3.799	−5.374	−2.224	−0.010	0.000
Iron Deficiency	−1.996	−3.524	−0.468	−0.006	0.010
Worm Infestation	−0.192	−1.627	1.243	−0.001	0.793
Fever	−0.045	−2.099	2.010	0.000	0.966
Cough	3.824	1.688	5.960	0.008	0.000
Inflammatory Diseases	−4.350	−6.460	−2.241	−0.009	0.000
Maternal Hemoglobin level (g/dl − 1 decimal)	0.740	0.733	0.747	0.421	0.000
Sex of child	1.857	0.702	3.011	0.006	0.002
Highest educational level	3.767	3.073	4.461	0.026	0.000
Respondent’s current age	0.393	0.276	0.510	0.014	0.000
Current age of child	−1.743	−2.204	−1.282	−0.016	0.000
Wealth index	1.459	0.952	1.967	0.014	0.000

**Table 6:** Mean Indices of hemoglobin level and associated factors.

Hemoglobin and associated factors	Number	Mean (95% CI)
Hemoglobin	215423	13.07 (12.453–13.687)
Vitamin A	247743	0.63 (0.626–0.634
Iron	247743	0.29 (0.286–0.294)
Height for age	236444	2.64 (−10.641–15.921)
Weigth for age	236444	1.86 (−9.553–13.273)

## Discussion

The analysis of the DHS 2015/2016 data revealed that nutritional intake, maternal health and educational statuses as well as household wealth are major determinants of childhood anemia in India. These raise questions regarding the implementation of the anemia-control policies that were put in place following the revelation of childhood anemia as a major challenge in the 2005/2006 survey. It also suggests the close links to maternal health status in India. Some scholars have suggested the possible existence of multiple pathways to the association between childhood anemia, iron status of children and their mothers^11^. For instance, antenatal anemia impacts on weight at birth and premature deliveries, a major risk factor in childhood anemia^16^. Severe anemia in mothers also impacts negatively on breast milk iron content leading to nutritional deficiency in the child. Iron intake among children in this population was generally low. All of these could act in concert to aggravate the anemic conditions in the children.

Mothers and their children share a common socioeconomic environment and realities, and as the child gets to12 months old, the dietary qualities of mother and child becomes essentially similar^11^. While child development may contribute to childhood anemia, some scholars failed to see any links between child development and childhood anemia^11,17^. Measurement of a child development trajectories could facilitate the identification of such an association. Unfortunately, birth records were not clearly presented in the data set.

We identified associations between childhood anemia and inflammatory infections. Other health conditions that tended to drive anemia in children included fever, cough and worm infestation. One obvious pathway of this relationship will be due to inappropriate iron losses. Pasricha, *et al*. argued that iron-deficiency anemia may thus be dependent on a complex interface of dietary-iron intake, increased iron use (growth velocity and erythroid mass expansion), and iron depletion due to parasitic infections and infestation^11^. Another finding that may be of interest to government and child survival programmes in India will be the level of Vitamin A intake among the population. This was often low and was associated with childhood anemia. A cue from this may be located in a successful government program to supplement vitamin A.

In this study important associations between household wealth and anemia were highlighted, suggesting direct influences of broader socioeconomic conditions directly on hemoglobin levels in children and hence childhood anemia in India^18,19^. This has been attributed to generalized bone marrow failure resulting from malnutrition, other micronutrients deficiencies, contact with biofuel smoke, and mechanisms linked to lower income and social statuses^11^.

According to Pasricha *et al*. some of the major effects of climate change are impairments of crop and agricultural yields, leading to food shortages and insecurity, challenge household wealth as well as childhood anemia^11^. Some also stress the point global financial crisis is a threat to the health status of countries in the low and middle-income strata^20^ and could drive childhood anemia through its effect on food insecurity and nutritional status of children. Consequently, childhood anemia may be aggravated when the conditions listed above are allowed to thrive and threaten socioeconomic realities of India populations. Combination of strategies to boost nutritional statuses of the people and address the social and economic conditions of the people may help mitigate the phenomena of childhood malnutrition and anemia^11,21^.

Scholars have also examined the etiology of anemia in children at various levels. A study of young children in Mexico demonstrated the preponderance of other cause related anemia over iron-deficiency related anemia. Infectious diseases and deficiencies in vitamin B and folate were more critical in explaining severe childhood anemia in Malawi^9^. Similarly, outcomes of researches in Thailand and USA also showed iron deficiency to be weak in explaining pediatric anemia^9,22,23^. Pasricha *et al*. studied the determinants of childhood anemia in India but this focused on in rural India. A study of 90 anemic children in urban slums of New Delhi investigated identified critical contributions of iron and vitamin B12 deficiency^11^.

The current study however, employed a large sample representative of rural, urban and urban slums of India. It confirmed findings from both the rural and urban slums of India. Further to confirmation of previous study findings, our analysis here highlights the biological, socio-demographic, and economic drivers of childhood anemia in India as a whole. Any differences between our findings on iron deficiency anemia and in previously published literature may reflect variations in diet and socioeconomic patterns in the different subsets of the populations and also in the analytical approaches.

In considering the outcomes of our study note should be taken of the following limitations. First, is that a cross-sectional study, the DHS unlike a longitudinal study can only support conclusion on association rather than causation. Second, the analysis was limited to variables contained in the DHS. Measurement of laboratory-based variables, and anthropometric measurement could have facilitated the detection of iron and vitamin deficiencies and of course strengthen the conclusions. Finally, recall on intake of iron and vitamin supplements in the survey may not be guaranteed thus introducing some limitations^24^ and could have engenders some overestimation of nutritional consumption in young children.

Despite these limitations, an all-inclusive set of factors linked to hemoglobin levels and childhood anemia in India has been identified. The representativeness of the large sample suggests the generalizability of the data to resource-limited settings in Asia. As a leading risk factor for childhood anemia in developing countries, iron deficiency anemia pose significant threat to impaired cognitive development in the children^1^. Universally, strategies promoting iron supplementation, food fortification, and dietary diversification have been promoted to fight against childhood anemia^25,26^.

While the Indian anemia control programme recommends the intake of iron and folic acid supplements among children younger than 5 years, the results here revealed that a failure of this programme in successfully controlling the occurrence of childhood anemia in Indian populations. This may be due to inefficient and sub-optimal programme implementation, poor adherence, among other causes. Further work on this is required to decipher the factors responsible for the gap between policies and practices regarding the control programmes mounted for anemia in this setting^27^. Our results therefore strengthen the call for a broad public health strategy for anemia control in Indian children beyond just delivering iron supplementation.

## Conclusions

Anemia is still prevalent among children in India and requires urgent attention. The results of the analysis of the DHS 2015/2016 data suggest that current efforts, namely iron and nutritional supplementation are necessary but not enough to significantly produce the necessary reduction in the incidence of childhood anemia. On the contrary, a combination of nutritional supplementation and food fortification programmes, as well as maternal anemia reduction efforts with alleviation of family poverty may translate into optimal improvement in the hemoglobin levels of the children.

## Declarations/Statements

- All methods in the Indian NFHS-4 2015/16 were carried out in accordance with relevant guidelines and regulation of conducting standard Demographic and Health Surveys and was monitored by the International Institute for Population Sciences, Deonar, Mumbai.
- The protocol for the collection of data under the NFHS-4 2015/16 was approved by the Indian Ethics Review Board and the Ministry of Health and Family Welfare
- The paper employed data collected with the high standards of ethical compliance in all Demographic and Health Surveys (DHS). In the collection of this data, the purpose of the study was explained to the respondents or the case of children to the parent/responsible adult, even before biomarker collection and to obtain their consent before collecting any blood samples. In order to ensure that these individuals can make an “informed” decision about whether or not to be tested for anemia, the DHS questionnaire included consent statements which were read to the respondent. The approach for obtaining consent differed slightly when the eligible individual is a child under age 6 years or an adolescent age 15–17 years. For this category of participants, consent was obtained of one of the child’s parents, and, in the absence of a parent, the consent of a responsible adult who is at least 18 years of age.
